# A Curriculum Challenge—The Need for Outcome (Competence) Descriptors

**DOI:** 10.3390/pharmacy5010007

**Published:** 2017-02-17

**Authors:** Ieva Stupans

**Affiliations:** School of Health and Biomedical Sciences, RMIT University, PO Box 71 Bundoora, Victoria 3083, Australia; ieva.stupans@rmit.edu.au

**Keywords:** accountability, communication, competencies, learning outcomes

## Abstract

Some outcomes around, for example, communication have been extensively theorised; others such as accountability have been relatively neglected in the teaching and learning literature. The question therefore is: if we do not have a clear understanding of the outcome, can we systematically apply good practice principles in course design such that students are able to achieve the outcomes the community and the profession expect? This paper compares and contrasts the literature around competency outcomes regarding students’ communication skills and the development of accountability and proposes a model to guide the selection of teaching and assessment approaches for accountability, based on the students’ sphere of influence.

## 1. Introduction

Our ability as educators to evaluate the effectiveness of our teaching depends, in part, on our ability to assess students’ learning. One of the key principles of good practice in curriculum design and in teaching is that of alignment between outcomes, learning opportunities and assessment. Suitable assessments can be designed only once standards for attainment have been clearly identified. The Competency Outcomes and Performance Assessment Model (COPA) provides a simple framework for competency-based or outcomes-based education. These are: (1) What are the essential competencies and outcomes for contemporary practice? (2) What are the indicators that define those competencies? (3) What are the most effective ways to learn those competencies? (4) What are the most effective ways to document that learners have achieved the required competencies [1]? The questions within this framework essentially capture the constructively aligned curriculum paradigm in which the desired learning outcomes are expressed in terms of activities students are required to be able to demonstrate, with teaching and learning activities and assessment being designed to be consistent with these desired learning outcomes [2]. The process of defining outcomes is critical as the outcomes determine the focus of learning and assessment; however, they also communicate external reference points at the national and international levels both within and outside the profession. An improvement in students’ being “able to do” allows the inference of the achievement of the desired learning outcomes and potentially the impact of our teaching.

In the health care literature, the terms competency, competencies, competence and competences are frequently used; these terms imply the ability to perform specific tasks, actions or functions successfully. The use of these terms also aligns with educational achievement by students, essentially a capacity or skill that is developed by the student. Competence is an outcome and, from the perspective of providing a program of study for students, sits within an outcome-oriented degree framework which refers to specific statements that describe what a student will be able to do in a measurable way. For the purposes of this paper the term *outcomes* will be used for both competence and learning outcome requirements. This is consistent with international standards and guidelines from the European Union [3], the United States Lumina Foundation Degree Qualifications Profile [4] and the Australian Qualifications Framework [5].

With the focus in higher education on preparing students for future employment, elements of a profession’s core competencies are normally incorporated into specified outcomes (i.e., competency-based learning outcomes) for that profession’s education programs. In the case of pharmacy programs, this process is well established, having been advocated in the 1997 World Health Organisation documents “The Role of the Pharmacist in the Health Care System” [6]. Anticipated end of degree outcomes for pharmacy graduates from Australia, Canada, the United Kingdom and the United States are all very similar and, with few exceptions, align well with the to the International Pharmaceutical Federation (FIP) Global Competency Framework [7]. With regard to the COPA model, essential outcomes for contemporary practice such as communication have been clearly outlined.

Learning outcomes are generally written with Bloom’s taxonomy in mind—Bloom’s taxonomy provides a framework for the process of learning whereby in the case of the cognitive domain, synthesis and evaluation represent the higher-order stages of thought processes. Similarly, in the affective domain, progress is demonstrated from a basic willingness to receive information for the integration of beliefs, ideas and attitudes. In the psychomotor domain, a number of taxonomies describe the development of skills and the coordination of brain and muscular activity [8]. With reference to the outcomes focused on in this paper, all three domains of Bloom’s taxonomy are relevant to communication: knowledge (cognition), motivation (affect) and skills (psychomotor abilities) [9]. Communication can be enhanced or diminished by any one of these components. Development of accountability aligns with the “continuum of internalisation” of affective values [8,10]. Assessment strategies depend on the domain of learning being assessed [11]. For example, the assessment of skill levels of communication needs to be based on actual performance. As students progress through a program of study, learning outcomes may be written such that a higher level of performance is progressively expected [8]. Learning outcomes should be clearly written, be assessable and be achievable [8].

The Dreyfus model has illuminated the developmental progression around skill acquisition and knowledge articulation embedded in expert practice [12]. This developmental model describes stages from novice, advanced beginner, competent, proficient to expert [13] and can be utilised to provide a framework for student progress towards a given outcome. The Association of Faculties of Pharmacy of Canada Educational Outcomes Task Force has utilised some of the features of the model to create descriptions of outcomes at three levels—below that required to graduate, graduation level and above expected level of performance [14]. For example, students performing at a level below that required to graduate “may use their communication skills in a formulaic manner or unstructured manner, resulting in inefficient use of time and potentially ineffective intervention”, whereas at a level above the expected level of performance they are able to “demonstrate an ease of communication that enables patients and other health care providers to rapidly develop trust and confidence in their professionalism and competence as a health care provider”. These levels can be used as the basis for the development of specific assessment tools.

Rubrics may be used to further illustrate to students the expectations of teaching staff around learning outcomes. Rubrics provide a coherent set of criteria for assessments for the learning outcome and descriptions of levels of performance quality for these criteria [15,16]. Rubrics have the potential to promote learning by making expectations and criteria for assessments of learning outcomes explicit [17]. The Association of American Colleges and Universities has developed VALUE (Valid Assessment of Learning in Undergraduate Education) rubrics [18] for 16 learning outcomes including the development of communication skills within programs. For example, Table 1 displays two criteria, one each for written and oral communication, and for novice to proficient performance.

## 2. Curriculum Design to Promote Outcomes around Communication

The COPA model requires that indicators for outcomes (competencies) are defined. A number of resources can be used to support academics in establishing standards for the attainment of outcomes concerning communication for their own university’s programs. These resources include guidelines from the European Union [3], which specifies that a cycle 1 graduate (essentially equivalent to bachelor’s degree) can communicate information, ideas, problems and solutions to both specialist and non-specialist audiences. The United States Lumina Foundation Degree Qualifications Profile [4] specify that at the bachelor’s level, the student is able to construct sustained, coherent arguments, narratives or explications of issues, problems or technical issues and processes, in writing and at least one other medium, to general and specific audiences. The Australian Qualifications Framework [5] specifies that graduates with a bachelor’s degree will have communication skills to present a clear, coherent and independent exposition of knowledge and ideas. Within individual programs VALUE rubrics [18] may also be adapted. These external resources can be used to promote a shared understanding of the standards for outcomes in an entire program of study.

## 3. Curriculum Design to Promote Outcomes around Communication in Pharmacy

The concept of the “the seven star pharmacist” developed over two decades ago proposed essential, minimum, common expectations of specific knowledge, attitudes, skills and behaviours for pharmacists. In the role of the pharmacist as a communicator, “He or she must be knowledgeable and confident while interacting with other health professionals and the public. Communication involves verbal, non-verbal, listening and writing skills” [6]. Communication skills are included in the more recently developed FIP Global Competency Framework as well as in outcome frameworks from a number of jurisdictions such as Australia, Canada, the United Kingdom, the United States [7] and the European Union [19,20]. A reported systematic search of pharmacy education literature identified that oral interpersonal communication skills and clinical writing skills were most often taught through simulated and standardised patient interactions and pharmacy practice experience courses with both subjective and objective assessments reported [21]

Identification of the relevant knowledge, skills, and attitudes that are pertinent to one aspect of communication for pharmacists, i.e., the therapeutic encounters between pharmacists and patients, has been facilitated through comparison to work in medical education which has defined elements which characterise effective communication in several clinical contexts [22], providing a coherent framework for assessing communication skills. For example, a single rubric which described four communication domains (structuring the encounter, establishes a trusting relationship, utilises effective verbal and nonverbal communication and retrieval and delivery of information) enabled the demonstration of longitudinally improving communication skills across five semesters of a pharmacy program [23].

## 4. Curriculum Design to Promote Outcomes around Accountability

In addition to being classified by profession-differentiated competencies, it has been suggested that all health professionals are defined as accountable practitioners [24] and indeed accountability is regarded as an essential competency of professionalism. However, accountability is an ambiguous term, often interchanged with responsibility. For the purposes of this paper accountability is defined as the continuous process of monitoring one’s professional conduct, through independent thought, explaining and justifying actions, whereas responsibility traditionally means performing tasks in an accurate and timely way [25].

Learning opportunities for and assessment of accountability have been relatively neglected in the teaching and learning literature and indicators for the achievement of accountability are highly varied. Accountability has been linked to something as simple as hand washing in routine clinical practice [26] or maintaining competence and undertaking continuing professional development [27].

For students, measurable indicators have yet to be refined as can be seen from an analysis of a cross-section of recent literature described below, which specifically references the learning of accountability.

- Professional conduct and accountability has been described as being strengthened [28] through a role play exercise in process engineering in which students worked in engineering production teams. Here accountability was identified through questioning of students on all aspects of the production process, presumably demonstrating team participation with students accepting responsibility for their statements and assertions.
- Students have been encouraged to be accountable participants in their learning and actively engage in self-directed learning through planning forms for clinical placements which were assigned grades [29]. Team-based learning with specific guidelines to nursing students around “readiness” to participate has also been associated with accountability demonstrated through advanced preparation for classes or contributions to team activities [30]. A similar strategy of requiring advanced preparation for classes in flipped classrooms, where materials are provided to students outside of formal class time and using formal class time for students to undertake collaborative and interactive activities, has also been specifically associated with developing students’ accountability [31].
- An enquiry-based training program for nursing students, collaboratively developed with a legal firm [32] which includes a simulated court case has been evaluated through student feedback, “Students felt that the module had strengthened their knowledge about accountable practice” [32] (p. 719), with further work from the same group substantiating the teaching approach [33].
- High-fidelity simulation cases which provide students with a realistic patient learning experience using computerised mannequins have been used to prompt nursing students to identify accountability skills and thus “may assist students in learning accountability”, [34] (p. 430).
- Engineering accountability has also been taught through physical prototyping of design projects, i.e., fabrication of designs rather than production of paper designs, which are tested and verified against project objectives with the outcome of “added accountability” [35].
- In physical therapy, a curriculum innovation which included a combination of standardised patients, reflection and online communities of practice in a 360-Degree assessment loop has been described as resulting in changes to student awareness of professional core values, including accountability. In this case, accountability, which included acknowledgement and acceptance of the consequences of one’s own actions, was self-assessed [36]. It is important to acknowledge that the examples cited in this paper are portions of a larger curriculum and no comment can be made regarding the accountability of the programs’ graduates.

## 5. Curriculum Design to Promote Outcomes around Accountability in Pharmacy

World Health Organisation guidelines on good pharmacy practice make clear reference to pharmacists as professionals with responsibilities and accountabilities which include “seeking to ensure that people derive maximum therapeutic benefit from their treatments with medicines” [37]. In the United States of America, hospital pharmacists have emphasised personal accountability for their professional practice as a unifying strategy for over 50 years [38]. In Australia the current competency framework also addresses accountability, for example “Pharmacists are accountable for the services provided and the associated outcomes” [39]. The professional competencies for Canadian pharmacists at entry to practice specify “accept responsibility and accountability for own actions and decisions” [40]. This core competency is also incorporated into outcomes for students. The American Association of Colleges of Pharmacy‘s Center for the Advancement of Pharmacy Education (CAPE) Educational Outcomes specify accountability to both professional practice and to patients [41]. Reliability, responsibility and accountability are defined in terms of being punctual, fulfilling responsibilities in a timely and manner, following instructions, undertaking activities in a self-directed manner, demonstrating a desire to exceed expectations, demonstrating accountability and accepting responsibility for one’s own actions [41]. Learning outcome statements from Australia and Canada both specify accountability towards patients [7]. As regards standards the Association of Faculties of Pharmacy of Canada Educational Outcomes Task Force provides, only one outcome description references accountability, and this is at a level below that required to graduate “violate fundamental ethical principles related to professional accountability” [14].

Learning opportunities for and the assessment of accountability in the pharmacy education literature have described team-based learning, promoted as holding students accountable for pre-class preparation [42,43]. Demonstration to students of accountability for professional actions through a patient advocacy–related curriculum using oral presentations and role play [44] has also been proposed, however not evidenced.

## 6. Refining Outcomes around Accountability

The literature which references the teaching and learning of accountability can be categorised in two different approaches. In the first of these approaches, students are “rewarded” explicitly through marks for the demonstration of accountability though preparation for learning activities [29,42,43], performance in teams [28,30] or adequate preparation for flipped classroom activities through low-stakes assessment [31], which do not relate specifically to professional practice. Teachers, rather than students, undertake the monitoring role and the actual transference of accountability to professional practice is unknown. “Training” is focused on the individual student being accountable to themselves, or to their team.

In the second approach, students participate in practical, simulated activities that “evoke or replicate substantial aspects of the real world” ([45] (p. i2), [44]). Learning of accountability is “evidenced” through students being able to identify accountability through these activities [32,33,34] or anticipated by academic staff [35]. No explicit reference is made to measure of achieving accountability [32,33,34,35] and, again, actual transference of accountability to professional practice post-graduation is unknown. In this case, students are exposed to concepts of accountability to their patients or clients and the community.

**Figure 1:**
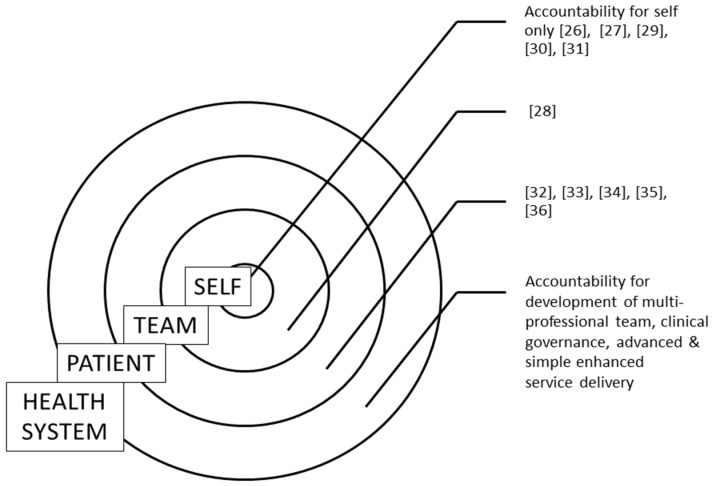
Examples of teaching and assessment of accountability from literature mapped according to sphere of influence.

Thus, although the curriculum has been described as being focused on accountability, it is in fact focused on accountability to the self, to the team or accountability to patients/clients. The curriculum examples cited in this paper are displayed according to the focus of the sphere of influence for curriculum innovation, i.e., self, team, patient/client and the broader health system, in Figure 1.

## 7. Discussion

Identification of appropriate indicators for the achievement of a desired competence is critical to being able to assess student outcomes. In the case of communication clear outcomes, teaching approaches and assessment are regularly described in the literature. Consideration by university teachers of the appropriate sphere of influence for a student will facilitate clarification of the outcome as accountability to the self, to the team, to patients/clients or indeed to the broader health system and the development of both teaching and assessment activities appropriate for each student cohort. This consideration means that the outcome accountability may be refined, for example, “students are accountable for pre-class preparation”, and learning activities and assessment consequently focused explicitly on accountability to the self.

## 8. Conclusions

Identifying appropriate teaching approaches and assessments depends upon the desired outcomes. This paper presents a comparison between the outcomes of accountability and communication. In the case of communication, outcomes are clearly defined and resources are available to inform teaching and assessment of communication. However, in the case of the critical outcome accountability, valid and reliable assessments and approaches to the teaching of accountability are yet to be developed. Figure 1 displays examples of accountability teaching and learning from the literature mapped according to the sphere of influence of an individual student. This paper adds to the literature by providing a model which may be useful for teaching staff considering teaching and assessment activities around the critical competence of accountability.

This paper has focused on outcomes as being central to students’ achievement. However, it is important to acknowledge that there are other factors which affect student learning and determine whether students develop the requisite outcomes, for example the approaches educators use to design and teach courses.

## Figures and Tables

**Table 1:** Descriptors for one written and one oral communication skill (sourced directly from Association of American Colleges and Universities [18]).

Criteria	Novice to Expert Categories	Descriptor
**Context of and Purpose for Writing *Includes considerations of audience, purpose, and the circumstances surrounding the writing task(s).***	Beginner: Students in the early stages	Demonstrates minimal attention to context, audience, purpose, and to the assigned tasks(s) (e.g., expectation of instructor or self as audience).
	Novice: Students in the middle stages	Demonstrates awareness of context, audience, purpose, and to the assigned tasks(s) (e.g., begins to show awareness of audience’s perceptions and assumptions).
	Competent: Graduates of this course	Demonstrates adequate consideration of context, audience, and purpose and a clear focus on the assigned task(s) (e.g., the task aligns with audience, purpose, and context).
	Proficient: Graduates as new professionals	Demonstrates a thorough understanding of context, audience, and purpose that is responsive to the assigned task(s) and focuses all elements of the work
**Delivery**	Beginner: Students in the early stages	Delivery techniques (posture, gesture, eye contact, and vocal expressiveness) detract from the understandability of the presentation, and speaker appears uncomfortable.
	Novice: Students in the middle stages	Delivery techniques (posture, gesture, eye contact, and vocal expressiveness) make the presentation understandable, and speaker appears tentative.
	Competent: Graduates of this course	Delivery techniques (posture, gesture, eye contact, and vocal expressiveness) make the presentation interesting, and speaker appears comfortable.
	Proficient: Graduates as new professionals	Delivery techniques (posture, gesture, eye contact, and vocal expressiveness) make the presentation compelling, and speaker appears polished and confident.
